# Exercise and physical activity interventions in type 2 diabetes mellitus

**DOI:** 10.3389/fendo.2026.1887543

**Published:** 2026-08-20

**Authors:** Weilong Su, Xinyue Li, Haicheng Gou, Lingfeng Yuan, Lixin Lin, Xiuting Lin, Xiaodong Wang, Yingzhe Xiong, Yisheng Luan

**Affiliations:** 1 Physical Education Institute, Putian University, Putian, China; 2 School of Physical Education and Sports, Central China Normal University, Wuhan, China; 3 Division of Sports Science and Physical Education, Tsinghua University, Beijing, China

**Keywords:** diabetes-related complications, exercise, glycemic control, physical activity, type 2 diabetes mellitus

## Abstract

Type 2 diabetes mellitus (T2DM) is among the most prevalent chronic noncommunicable diseases worldwide and imposes a substantial burden on public health. Exercise and physical activity are cornerstones of T2DM management, but current recommendations remain largely centered on standardized exercise doses and uniform physical activity targets, with limited consideration of individual variability and medication-related safety. This review integrates current mechanistic, clinical, and guideline-relevant evidence on exercise and physical activity interventions in T2DM, with the aim of clarifying how these may be applied more appropriately across different clinical contexts. Different exercise modalities provide distinct benefits for glycemic control, physical function, and cardiometabolic health, indicating that exercise modality and dose should be selected according to the dominant therapeutic objective. Beyond modality and dose, exercise timing may further influence metabolic responses and should be considered when designing structured exercise programs. Interrupting prolonged sitting should also be incorporated as a complementary behavioral target alongside planned exercise. Because exercise is commonly implemented alongside glucose-lowering therapy, medication-related safety should also be considered when individualizing exercise programs. In individuals with diabetes-related complications, exercise should not be regarded as universally contraindicated, but should be adapted according to complication severity, safety considerations, and functional capacity. Older adults require prescriptions that preserve muscle strength, balance, joint mobility, and functional independence, whereas youth-onset T2DM requires early, developmentally tailored strategies that support long-term adherence. Overall, exercise and physical activity should be conceptualized as a precise, adaptable, and clinically essential therapy for T2DM.

## Graphical Abstract

**Figure:**
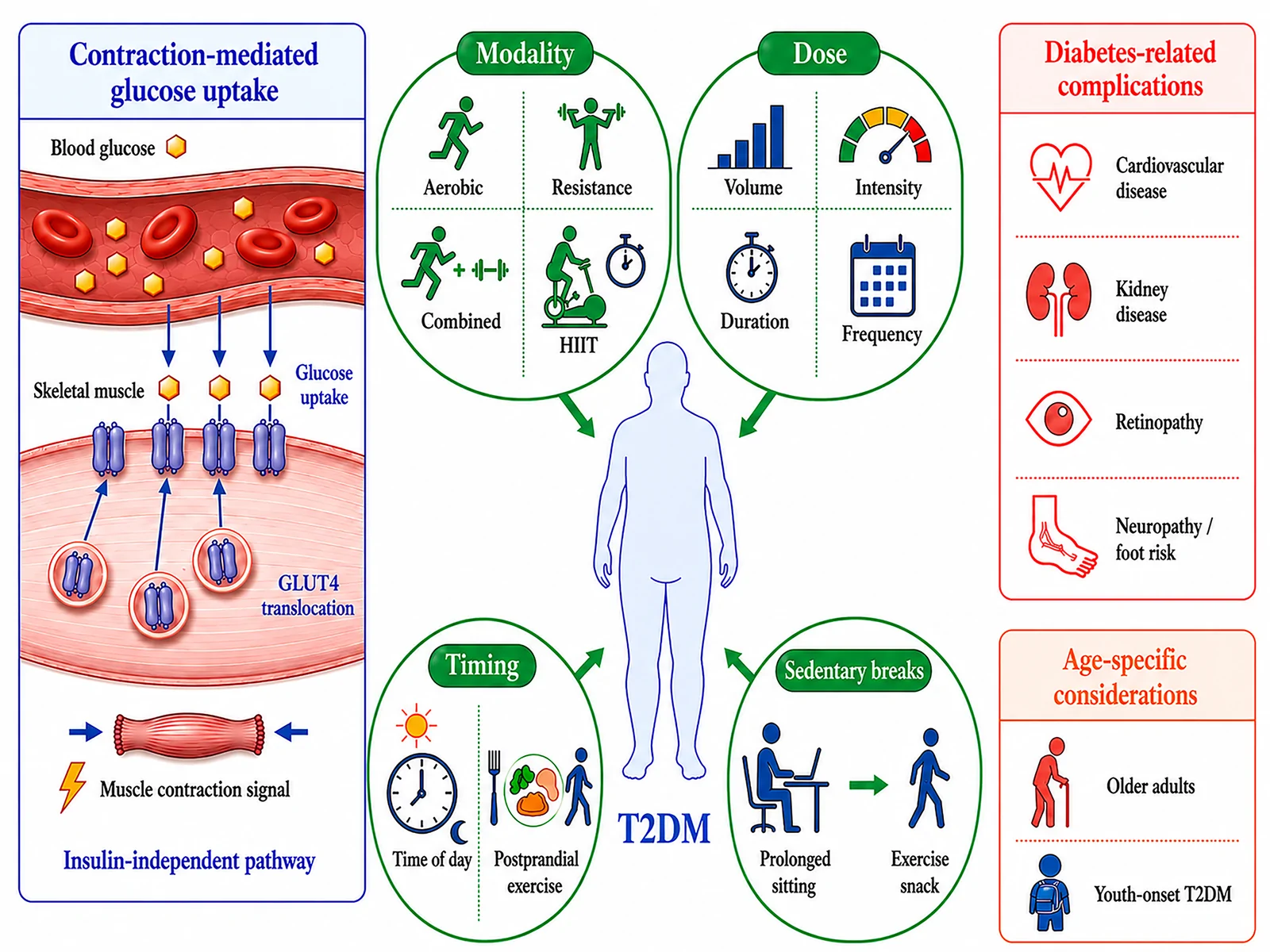

## 1 Introduction

As one of the most prevalent chronic noncommunicable diseases worldwide, diabetes poses a major global public health challenge. In 2024, approximately 589 million adults aged 20–79 years were living with diabetes globally, representing 11.1% of adults in this age group, and this number is projected to reach 852.5 million by 2050 (1). Type 2 diabetes mellitus (T2DM), which accounts for more than 90% of diabetes cases worldwide, is characterized by persistent hyperglycemia resulting from insulin resistance and insufficient compensatory insulin secretion (2). Chronic hyperglycemia and related metabolic disturbances contribute to progressive microvascular and macrovascular complications, making sustained glycemic control and cardiometabolic risk reduction central goals of T2DM management.

Because T2DM is a chronic condition requiring long-term management and pharmacological therapy primarily controls rather than reverses the underlying metabolic disorder, sustainable lifestyle-based strategies are essential to its care. Exercise is a cornerstone of T2DM management, with established benefits for glycemic control, insulin sensitivity, cardiorespiratory fitness (CRF), physical function, and cardiometabolic health. Current ADA and ACSM guidance provides an established framework for exercise management in T2DM and recognizes the need for individualized implementation (3–5). However, evidence continues to evolve regarding how the effectiveness, safety, and feasibility of exercise and physical activity interventions vary across different clinical contexts.

Accordingly, this narrative review synthesizes current mechanistic and clinical evidence on exercise and physical activity interventions in T2DM. It examines acute and chronic effects on glucose homeostasis, exercise modalities, weekly exercise dose, exercise timing, interruption of prolonged sedentary behavior, medication-related considerations, diabetes-related complications, and age-specific factors. By interpreting emerging evidence alongside established guideline recommendations, the review aims to provide a structured evidence overview that clarify the clinical application of exercise and physical activity interventions across different clinical contexts and informs future intervention design.

## 2 Literature search and evidence selection

We searched PubMed and Web of Science to identify relevant literature. No publication-date restrictions were applied, and the final search was conducted on 2 April 2026. Search terms included combinations of “type 2 diabetes” or “T2DM” with terms related to exercise, physical activity, exercise modality, exercise timing, sedentary behavior, diabetes-related complications. Full search strings, date limits, restrictions, and screening details are provided in **Supplementary Table 1**. Two reviewers independently screened the titles, abstracts, and full texts of potentially relevant articles, and any disagreements were resolved through discussion. We prioritized recent and high-quality evidence, including clinical guidelines, consensus statements, systematic reviews, meta-analyses, randomized controlled trials, and mechanistic studies. Reference lists of key articles were manually screened to identify additional relevant studies. Other study designs were cited when evidence on a specific topic was limited. Relevant narrative reviews were included when they provided important mechanistic or clinical context, or when no recent systematic review was available. Given the narrative nature of this review and the restriction of the literature search to PubMed and Web of Science, some degree of author judgment in evidence selection was inherent, and relevant studies indexed exclusively in other databases may have been missed.

## 3 Acute and chronic exercise effects on glucose homeostasis

### 3.1 Acute effects of a single exercise bout

During exercise, skeletal muscle increases glucose uptake through insulin-independent mechanisms that regulate glucose delivery, transport across the muscle-cell surface, and intramyocellular metabolism (6). Exercise increases glucose delivery by augmenting muscle blood flow and capillary recruitment (7). At the same time, muscle contraction stimulates glucose transporter type 4 (GLUT4) translocation to the sarcolemma and T-tubules, thereby increasing glucose transport into the myofiber (8, 9). Once glucose enters the myofiber, it is phosphorylated by hexokinase II to glucose-6-phosphate, which maintains the transmembrane glucose gradient and facilitates further glucose uptake (10, 11). In parallel, acute exercise also increases hepatic glucose output, which helps maintain blood glucose homeostasis by matching hepatic glucose release to the increased glucose demands of contracting skeletal muscle (12).

**Figure 1:**
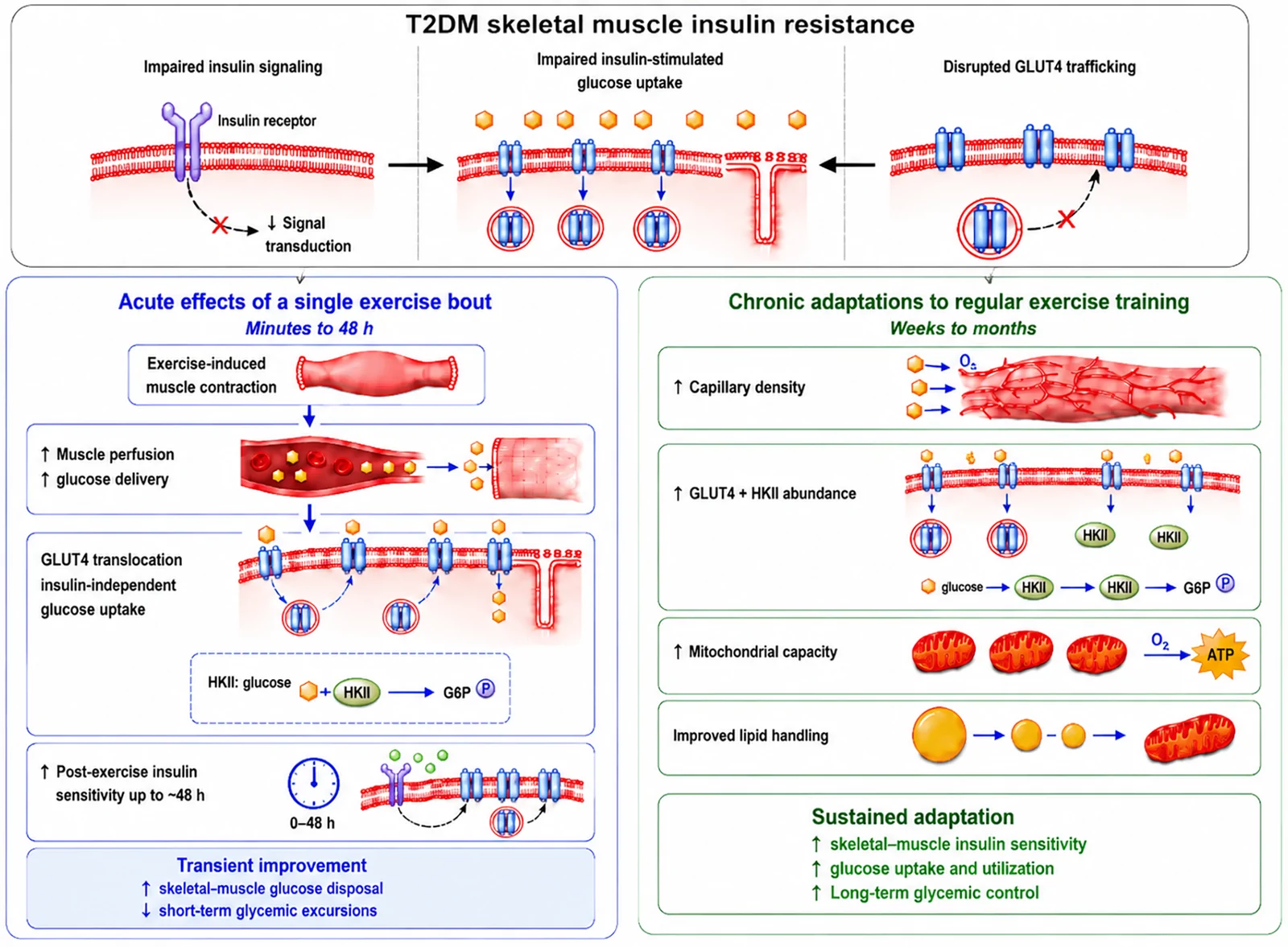
Acute responses and chronic adaptations of exercise for glucose homeostasis in T2DM. A single exercise bout increases muscle perfusion, promotes insulin-independent GLUT4 translocation, and enhances post-exercise insulin sensitivity for up to approximately 48 h. Regular exercise training induces sustained adaptations, including increased capillary density, greater GLUT4 and HKII abundance, enhanced mitochondrial capacity, and improved lipid handling, thereby supporting skeletal muscle glucose uptake and long-term glycemic control.

In T2DM, skeletal muscle represents a major site of insulin resistance, where impaired insulin-stimulated glucose uptake contributes to whole-body glucose dysregulation (13). This insulin-resistant defect is associated with impaired insulin signaling and disrupted GLUT4 trafficking, resulting in reduced insulin-stimulated GLUT4 delivery to the sarcolemma and T-tubules (14). Mechanistically, microtubule- and kinesin-1/KIF5B-dependent GLUT4 movement appears to maintain an insulin-responsive GLUT4 pool for recruitment to the sarcolemma and T-tubules, and this process is impaired in insulin-resistant muscle models (15) (**Figure 1**). Against this background, the contraction-mediated pathway is particularly relevant in T2DM because exercise-induced skeletal muscle glucose uptake and GLUT4 translocation are not impaired, thereby providing an insulin-independent mechanism that supports the glucose-lowering efficacy of exercise in this population (16). Human skeletal muscle biopsy data support this interpretation, showing that a single bout of moderate cycling increased plasma membrane GLUT4 content in individuals with T2DM by approximately 74%, a relative increase comparable to that observed in control participants (17). Together, these findings indicate that a single exercise bout can acutely enhance skeletal muscle glucose disposal in T2DM through contraction-mediated, insulin-independent mechanisms. Beyond these immediate effects during exercise, a single exercise bout also enhances insulin sensitivity during recovery, with increased insulin-stimulated glucose disposal and skeletal muscle glucose uptake for up to 48 h (18, 19).

### 3.2 Chronic adaptations to regular exercise training

Although a single exercise bout can acutely improve glucose uptake and insulin sensitivity, these metabolic benefits are transient and are rapidly lost without continued exercise (20). In contrast, chronic exercise drives longer-term adaptations that improve whole-body and skeletal muscle insulin sensitivity by increasing capillary density, augmenting GLUT4 and hexokinase II abundance, enhancing mitochondrial volume and respiratory capacity, and modulating intramyocellular lipid accumulation and lipid droplet size (16, 20) (**Figure 1**). Collectively, these structural and metabolic adaptations increase the capacity of skeletal muscle to take up and utilize glucose and thereby contribute to improvements in glycemic control. Notably, the magnitude of improvement in insulin sensitivity may be influenced by exercise duration, independent of exercise intensity and volume (21, 22).

## 4 Exercise and physical activity strategies in T2DM

### 4.1 Exercise modalities

#### 4.1.1 Aerobic training

Aerobic exercise has long been regarded as a principal exercise modality in the management of T2DM. Aerobic exercise not only improves glycemic control but also enhances CRF and overall cardiometabolic health (23). Current guidelines and consensus statements consistently recommend that adults with T2DM engage in at least 150 min/week of moderate-intensity aerobic exercise, performed on at least 3 days per week and with no more than 2 consecutive days without exercise (3–5, 24). This frequency recommendation is based on the insulin-sensitizing effects of exercise, which may persist for up to approximately 48 h after the last exercise (18, 19). However, dose-response evidence from supervised aerobic exercise indicates that each additional 30 min/week reduces glycated hemoglobin (HbA1c) by 0.22 percentage points, with the dose-response curve beginning to flatten at approximately 100 min/week (25). In previously inactive individuals, aerobic exercise should be performed for at least 10 min, with the goal of accumulating approximately 30 min or more on most days of the week, so as to achieve at least 150 min/week (3, 5). Consistent with this, evidence from meta-analyses suggests that progressive aerobic training is associated with greater improvements in HbA1c than non-progressive aerobic training, supporting the importance of gradual progression in exercise prescription for glycemic management in T2DM (25, 26).

Several aerobic training variants have been investigated as alternatives to conventional continuous aerobic exercise. Interval walking training, consisting of alternating 3-min bouts of fast walking at approximately 70%–90% of VO_2_peak and slow walking at approximately 40%–60% of VO_2_peak, may improve glycemic control and insulin sensitivity and reduce glycemic variability more effectively than energy expenditure-matched continuous walking in adults with T2DM (27–29). Aquatic aerobic exercise represents another practical alternative, particularly for individuals with obesity, osteoarthritis, joint pain, or impaired balance. Meta-analytic evidence showed that aquatic training improved HbA1c, peak oxygen uptake (VO_2_peak), blood pressure (BP), and 6-min walk distance in patients with T2DM, with effects on HbA1c, VO_2_peak, and BP broadly comparable to land-based training (30). Other alternative aerobic modalities, such as Nordic walking and aerobic dance, may provide additional options for individuals with T2DM. However, the current evidence remains relatively limited and heterogeneous, and does not yet support these modalities as standard alternatives to conventional aerobic training.

#### 4.1.2 Resistance training

Diabetes is an independent risk factor for reduced muscle strength and impaired physical function (31). In adults with T2DM, resistance exercise training is associated with improvements in muscle strength, skeletal muscle mass, cardiometabolic risk factors, and insulin sensitivity (32–35). The American College of Sports Medicine (ACSM) and American Diabetes Association (ADA) recommend resistance training at moderate (50%–69% of one-repetition maximum [1RM]) to vigorous (70%–85% 1RM) intensity on at least 2–3 nonconsecutive days per week (3, 4). Training should begin at a moderate-intensity (10–15 repetitions per set), with progression achieved by increasing the load first, followed by the number of sets, and finally training frequency (3, 4). Resistance training intensity should not be defined solely by a nominal percentage of 1RM. Although direct 1RM testing is generally reliable, it may be impractical or inappropriate in older, frail, or complication-burdened adults with T2DM. When maximal testing is not feasible, training load can be prescribed and progressed using estimated 1RM derived from submaximal multiple-repetition testing, repetitions-in-reserve (RIR)-guided autoregulation, rating of perceived exertion (RPE)-based monitoring, or velocity-based methods when equipment is available (36–38). However, in older adults, RIR and RPE should be interpreted cautiously because perceptual accuracy may be limited (38). Importantly, the training stimulus depends not only on the nominal %1RM but also on proximity to failure, particularly during low-load resistance exercise, where sets often need to be performed closer to failure to provide a meaningful stimulus (39).

In terms of glycemic outcomes, meta-regression analysis suggested that greater improvements in muscular fitness may be associated with larger reductions in HbA1c, although the association was only borderline statistically significant (β = −0.99, 95% CI −1.97 to −0.01; p = 0.047) (40). Multiple network meta-analyses have suggested that aerobic exercise is more effective than resistance exercise in reducing HbA1c (41–43). However, this relationship may vary by body weight status, as a randomized controlled trial in normal-weight individuals with T2DM showed a greater reduction in HbA1c with resistance training alone than with aerobic training alone (44). Emerging evidence further suggests that low-load blood-flow restriction resistance training (BFR-RT) may represent a practical and safe alternative to conventional resistance training for individuals with T2DM who have musculoskeletal limitations or reduced functional capacity (45–48). A typical BFR-RT prescription uses low external loads of approximately 20%–40% of one-repetition maximum, with cuff pressure individualized to limb occlusion pressure, commonly about 40%–80% of limb occlusion pressure rather than a fixed absolute pressure (45, 49, 50). Despite a markedly lower mechanical load, BFR-RT elicits comparable improvements in muscle strength and hypertrophy, while additionally improving mitochondrial oxidative capacity in skeletal muscle and adipose tissue and reducing visceral adipose tissue volume (45–48). However, BFR-RT should not be recommended broadly for patients with complicated T2DM. Given the potential cardiovascular, vascular, and thrombotic concerns associated with BFR, it requires pre-participation screening and individualized cuff-pressure determination, and should be avoided or used only after specialist clearance in patients with severe non-proliferative or proliferative diabetic retinopathy, uncontrolled hypertension, active or recent venous thromboembolism, major bleeding or clotting disorders, severe peripheral vascular disease, or unstable cardiovascular disease (48–51).

#### 4.1.3 Combined training

Combined aerobic and resistance training has emerged as a particularly effective strategy for the management of T2DM. Multiple network meta-analyses suggest that combined training provides greater improvements in HbA1c than either aerobic training alone or resistance training alone (41–43, 52). Importantly, its advantages extend beyond glycemic control. Compared with single-modality exercise, combined training provides a broader overall benefit profile, with concurrent improvements in CRF, muscular strength, body composition, and lipid-related outcomes (53). Practically, combined training integrates aerobic and resistance exercise within the same program, thereby providing complementary benefits while avoiding the limitations of a single exercise modality. Dose-response meta-analysis suggests that, in adults with T2DM, the optimal weekly dose of combined training for improving HbA1c is approximately 1030 metabolic equivalent of task minutes per week (MET-min/week), corresponding to about 270 min/week of moderate-intensity or 150 min/week of vigorous-intensity training, with a minimum effective dose of approximately 370 MET-min/week (54).

Exercise order within a concurrent training session is a relevant programming consideration. Meta-analyses indicate little or no effect of within-session order on VO_2_max, whereas resistance-before-aerobic training provides a small advantage for lower-limb strength (55, 56). In T2DM, available studies suggest that both exercise orders improve glycemic control and cardiometabolic outcomes, with no consistently superior sequence (57–59). At the molecular level, the classic interference hypothesis proposes that endurance exercise-induced AMPK activation may inhibit mTORC1-mediated anabolic signaling. However, available human evidence does not support a simple linear antagonism, as AMPK activation can occur without suppression of subsequent resistance-exercise-induced mTORC1/S6K1 signaling, while different exercise sequences have shown broadly similar responses across these pathways (60–63). How the two exercise modalities are distributed across sessions is another practical consideration. Concurrent training does not appear to compromise maximal strength or muscle hypertrophy overall (64). However, explosive-strength gains may be attenuated with same-session programming, whereas this attenuation is not evident with separate-session programming when aerobic and resistance exercise are separated by ≥3 h (64).

#### 4.1.4 High-intensity interval training

High-intensity interval training (HIIT) refers to repeated high-intensity, but not all-out, exercise bouts separated by recovery periods and should be distinguished from sprint interval training (SIT), which involves all-out or supramaximal sprint efforts. For cardiorespiratory exercise, high intensity is physiologically defined as exercise performed above the second metabolic threshold but below the work rate associated with the attainment of VO_2_max during a graded exercise test (65). Current evidence suggests that HIIT ranks among the more effective exercise modalities for reducing HbA1c (42, 43, 66). The ADA Standards of Care in Diabetes suggest that, for more physically fit adults with T2DM, 75 min/week of vigorous-intensity or interval training may be sufficient as a practical guideline target (5). Notably, dose-response meta-analytic evidence suggests that HIIT may significantly reduce HbA1c at approximately 320–330 MET-min/week, equivalent to about 45 min/week of main HIIT session time, including recovery intervals but excluding warm-up and cool-down (66).

However, the applicability of HIIT may be limited in individuals with lower baseline fitness, greater symptom burden, or diabetes-related complications, and its long-term adherence in routine clinical practice remains insufficiently established. Lower-impact walking-based HIIT may partly address these feasibility concerns. In middle-aged and older adults with T2DM, treadmill walking HIIT using repeated 3-min bouts at 70% heart-rate reserve (HRR) interspersed with 3-min active recovery at 30% HRR improved acute glycemic control more than continuous walking at 50% HRR (67). Thus, low-volume walking-based HIIT may be a feasible interval-based option for selected lower-fitness individuals, but it should be introduced gradually and individualized according to cardiovascular risk, fitness level, and glycemic safety. In addition, because HIIT may induce transient post-exercise hyperglycemia, individuals with T2DM should be advised to monitor their glycemic responses closely (4).

### 4.2 Weekly exercise dose

Although current guidelines recommend weekly exercise targets for people with T2DM, recent studies examining the minimum effective and optimal weekly exercise dose for glycemic improvement have yielded inconsistent findings. One dose-response network meta-analysis estimated that a significant improvement in glycemic control emerges at approximately 840 MET-min/week and that additional benefit becomes markedly attenuated beyond about 1300 MET-min/week (66). In contrast, another meta-analysis identified 1100 MET-min/week as the optimal weekly dose and further suggested that the minimum effective dose required may differ according to baseline HbA1c (68). Taken together, both the minimum effective dose and the optimal dose range are context dependent rather than fixed, and should be interpreted in light of patient characteristics.

### 4.3 Exercise timing

#### 4.3.1 Time-of-day effects of exercise

In T2DM, circadian rhythmicity is often disrupted, a dysregulation that may be driven by insulin resistance, mitochondrial dysfunction, and chronic inflammation (69). This circadian disruption is characterized by relatively better insulin sensitivity and glycemic control in the evening, but poorer glucose regulation overnight and in the early morning, contributing to morning hyperglycemia, known as the dawn phenomenon (70). Exercise may serve as a non-photic zeitgeber that helps entrain circadian rhythms, suggesting that the glycemic responses to exercise may be influenced by the time of day at which it is performed (69). Accordingly, exercise timing has gained increasing attention as a potential modifier of glycemic responses, prompting clinical studies that compare morning with afternoon or evening exercise in individuals with T2DM.

Most studies suggest that morning exercise at moderate- to high-intensity may increase glycemia, whereas exercise of similar intensity performed in afternoon or evening is more often associated with a more favorable glycemic response and greater insulin sensitivity (71–74). However, a recent randomized controlled trial found no difference in 24-hour glucose profiles between morning and afternoon HIIT, although morning HIIT increased glucose during the 2-hour post-exercise period in adults with T2DM, whereas afternoon HIIT did not produce this rise (75). Likewise, continuous glucose monitoring showed that 50 min of treadmill walking at an average intensity of approximately 3.7 METs, performed in the morning, afternoon, or evening produced no significant differences in 24-hour mean glucose, glycemic variability, or postprandial glucose in adults with T2DM, despite a greater glucose decline during afternoon exercise (76). In longer-term training, a 12-week randomized trial found that supervised morning and evening multimodal exercise produced similar improvements in HbA1c, fasting glucose, postprandial glucose, and insulin resistance in overweight adults with or without T2DM (77). Taken together, later-day exercise may confer glycemic advantages in selected settings, especially for moderate- to high-intensity protocols, but current evidence does not support a universal exercise timing recommendation for all individuals with T2DM.

#### 4.3.2 Exercise timing relative to meals

In addition to time-of-day effects, the timing of exercise relative to meal ingestion may also influence glycemic regulation in T2DM. Current evidence suggests that postprandial exercise is more effective than preprandial exercise for improving acute postprandial glycemic control in individuals with T2DM (4, 78–80). Exercise initiated in the early postprandial period, typically within the first postprandial hour, is particularly effective in attenuating postprandial glucose elevations, likely because it increases skeletal muscle uptake of circulating glucose during the rising phase of postprandial glycemia, thereby blunting the postprandial glucose peak (81–83). By contrast, exercise performed later in the postprandial period still lowers blood glucose, but it may be less effective in attenuating the postprandial peak (79). Moreover, the ACSM consensus statement further notes that greater energy expenditure during postprandial exercise is associated with lower glycemia regardless of exercise intensity or modality and that exercise lasting at least 45 min provides the most consistent glucose-lowering effect (4).

## 5 Sedentary behavior and breaking up sitting

One study found that each additional hour of daily sedentary time was associated with 22% higher odds of T2DM, independent of body mass index (BMI) and higher-intensity physical activity (84). Among individuals with T2DM, greater sedentary time was associated with higher clustered metabolic risk (85). Mechanistically, prolonged sedentary behavior may impair skeletal muscle glucose uptake and disposal by reducing contractile activity. Potential molecular mechanisms include reduced skeletal muscle GLUT4 protein content, attenuated Akt-mediated insulin signaling, and reduced glycogen synthase activity, collectively contributing to lower skeletal muscle insulin sensitivity and worsening insulin resistance (86). Frequent activity breaks from sitting acutely stimulate contraction-mediated glucose uptake signaling and, when repeated over several days, may shift skeletal muscle toward greater insulin-signaling responses and enhanced glucose transport capacity. Specifically, in overweight sedentary adults, acute 2-min moderate-intensity walking breaks, performed at a Borg RPE of 12–14 and 5.8–6.4 km/h every 20 min, were associated with greater capacity for glycogen synthesis and adenosine triphosphate production (87). Such brief and intermittent bouts of physical activity have been conceptualized as exercise snacks, a time-efficient strategy for interrupting prolonged sitting. In adults with T2DM, interrupting prolonged sitting with 6-min bouts of simple resistance activity every 60 min reduced the whole-day glucose and insulin responses to meals by 21% and 13%, respectively, compared with uninterrupted sitting (88). Compared with approximately energy-matched structured cycling at 50%–60% of maximal workload capacity, breaking up sitting with standing and self-perceived light-intensity walking produced a greater improvement in insulin sensitivity, while achieving similar improvements in 24-hour glucose levels, in individuals with T2DM (89). From a clinical perspective, breaking up sitting may offer a practical long-term strategy because simple activity breaks can be incorporated into daily routines without requiring a dedicated exercise session.

## 6 Medication-related considerations for exercise interventions

In clinical practice, exercise interventions in T2DM are usually implemented alongside glucose-lowering pharmacotherapy. However, because glucose-lowering agents differ in their effects on insulin availability, glucose regulation, fluid balance, and ketone metabolism, background pharmacotherapy may modify both the glycemic response and the safety profile of exercise.

Exercise-related hypoglycemia is mainly a concern in individuals treated with insulin or insulin secretagogues, such as sulfonylureas and meglitinides (90–92). During exercise, skeletal muscle glucose uptake increases through contraction-mediated mechanisms (6, 7). Under physiological conditions, endogenous insulin secretion decreases, whereas hepatic glucose production increases to match the higher glucose demand of working muscle (93). This balance may be disrupted in patients using exogenous insulin or insulin secretagogues. Exogenous insulin action cannot be rapidly reduced at exercise onset, and insulin secretagogues may continue to stimulate endogenous insulin release despite increased glucose demand (3, 94, 95). As a result, skeletal muscle glucose uptake increases, whereas hepatic glucose output may not rise adequately under continued insulin action, thereby increasing the likelihood of exercise-related hypoglycemia. This risk may extend into recovery, because a single bout of moderate exercise can enhance post-exercise insulin sensitivity (18, 19). Accordingly, in individuals treated with insulin or insulin secretagogues, prolonged or late-day moderate-intensity exercise may increase the risk of delayed or nocturnal hypoglycemia (3). By contrast, vigorous or interval-based exercise may produce transient post-exercise hyperglycemia (4). In insulin-treated patients, excessive insulin reduction or unnecessary carbohydrate intake may worsen post-exercise hyperglycemia, whereas overcorrection of transient post-exercise hyperglycemia with insulin may increase the risk of delayed hypoglycemia (90). Current ADA recommendations and the ACSM consensus statement indicate that individuals using insulin and/or insulin secretagogues may require carbohydrate supplementation, clinically guided insulin adjustment, and glucose monitoring around exercise to reduce hypoglycemia risk (3–5).

For glucose-lowering agents with a low intrinsic risk of hypoglycemia, routine dose reduction around exercise is generally unnecessary. However, some may still modify training responses. Metformin is a notable example, as accumulating evidence suggests that it may modify selected cardiorespiratory, vascular, and metabolic adaptations to structured exercise. A meta-analysis involving 827 adults with glucose dysregulation found that adding metformin to structured exercise modestly attenuated improvements in VO_2_peak and reductions in systolic and diastolic BP, without significant differences in glycemic or lipid outcomes (96). In older adults, metformin also attenuated gains in cardiorespiratory fitness and whole-body insulin sensitivity and abolished the training-induced increase in skeletal-muscle mitochondrial respiration (97). Preclinical and translational evidence further suggests that metformin attenuated training-induced mitochondrial respiration and reduced exercise-responsive transcriptional programs related to oxidative capacity, myogenesis, proteostasis, and angiogenesis (98). A recent trial further suggested that this blunting effect may be particularly relevant during high-intensity training, as metformin was associated with smaller improvements in clamp-derived insulin sensitivity and insulin-stimulated carbohydrate oxidation (99). Nevertheless, these effects are not uniform across studies. Metformin was associated with greater lactate accumulation during exercise but did not consistently reduce VO_2_peak or VO_2_max, with responses varying according to dose, treatment duration, exercise intensity, and participant characteristics (100). These findings may be relevant when interpreting heterogeneous training responses, but they are insufficient to support withholding metformin or making exercise-specific dose adjustments.

Exercise interventions in patients receiving glucagon-like peptide-1 receptor agonists (GLP-1RAs) should be designed with attention to the clinical characteristics of this therapy. In adults with T2DM, GLP-1RAs improve glycemic control and produce clinically meaningful weight loss (101, 102). Several GLP-1RAs have demonstrated cardiovascular benefits, and selected GLP-1-based therapies have shown kidney benefits in high-risk populations (101, 103). Gastrointestinal adverse events, particularly nausea, diarrhea, constipation, and vomiting, are the most frequent adverse effects of GLP-1RAs and are typically most pronounced during treatment initiation or dose escalation, often attenuating over subsequent weeks (101). These symptoms may reduce exercise tolerance or adherence and should be considered when designing exercise interventions. Because GLP-1-based weight loss may be accompanied by reductions in lean mass, resistance training should be incorporated to preserve muscle quantity, strength, and physical function (102, 104). Clinical evidence suggests that structured exercise and GLP-1RAs have complementary effects on body composition and cardiometabolic risk markers, including greater reductions in body-fat percentage, abdominal adiposity, and hsCRP than either approach alone (104–106).

Sodium-glucose cotransporter 2 (SGLT2) inhibitors are widely used in adults with T2DM, particularly in those with coexisting heart failure or chronic kidney disease, because their benefits extend beyond glucose lowering to cardiovascular and renal protection (107, 108). However, they introduce a distinct exercise-related safety concern because of their association with diabetic ketoacidosis (DKA). A meta-analysis reported that, compared with placebo or other glucose-lowering therapies, SGLT2 inhibitor use was associated with relative risks for DKA of 2.46 in randomized trials and 1.74 in observational cohorts (109). By inhibiting glucose reabsorption in the renal proximal tubule, SGLT2 inhibitors increase urinary glucose loss and lower plasma glucose, which may reduce glucose-stimulated insulin secretion and increase the glucagon-to-insulin ratio, thereby promoting lipolysis and hepatic ketogenesis. Glycosuria, together with natriuresis, also promotes osmotic diuresis and volume depletion, which may amplify counter-regulatory responses and further enhance ketone production (108, 110, 111). Because urinary glucose excretion persists during ketosis, clinically significant ketonemia and metabolic acidosis may develop without marked hyperglycemia, a presentation known as euglycemic diabetic ketoacidosis, which can delay recognition (110–112). Prolonged or unusually strenuous exercise has been identified as a potential precipitant of SGLT2 inhibitor-associated DKA, particularly when combined with dehydration, low-carbohydrate intake, acute illness, prolonged fasting, or inappropriate insulin dose reduction or omission (112, 113). Therefore, patients should maintain adequate fluid and carbohydrate intake during prolonged exercise and should avoid inappropriate reduction or omission of insulin. The development of nausea, vomiting, abdominal pain, dyspnea, unusual fatigue, or malaise during or after exercise should prompt immediate cessation of exercise and assessment of blood ketones.

## 7 Exercise and physical activity in T2DM with diabetes-related complications

### 7.1 Cardiovascular disease and cardiometabolic risk

Cardiovascular disease (CVD) is a leading cause of morbidity and mortality in individuals with T2DM, who have a twofold to fourfold higher cardiovascular risk than adults without diabetes, with risk increasing as glycemic control worsens (114–116). Observational evidence consistently shows that higher levels of physical activity are associated with lower risks of CVD morbidity and mortality in T2DM (117–121). A dose-response meta-analysis involving 109,820 individuals with diabetes further showed that each 600 MET-min/week increase in physical activity was associated with a 19% lower risk of CVD morbidity and a 6.9% lower risk of CVD mortality (121). However, these findings support an epidemiological association between higher habitual physical activity and lower CVD risk, but they do not establish a causal effect of increasing physical activity on cardiovascular events. Evidence from randomized interventions remains less definitive. In adults with T2DM, the Look AHEAD trial showed that an intensive lifestyle intervention targeting weight loss through caloric restriction and increased physical activity did not significantly reduce cardiovascular morbidity or mortality during a median 9.6 years of intervention (122). Interventional evidence on whether exercise training reduces cardiovascular endpoints in adults with T2DM remains limited, partly because such endpoint trials require large sample sizes and extended follow-up. Most existing exercise intervention studies have focused on intermediate cardiometabolic risk markers that may help explain the epidemiological association between higher physical activity and lower CVD risk, including CRF, BP, vascular function, and lipid metabolism (123, 124).

CRF is a strong and independent marker of risk for cardiovascular and all-cause mortality (125). In adults with T2DM, exercise-induced improvements in CRF are associated with favorable changes in cardiovascular risk factors, independently of weight loss (126). Aerobic-based exercise, with or without resistance training, is among the most consistently supported strategies for improving CRF in adults with T2DM (127). Recent evidence suggests that HIIT may induce greater improvements in CRF than aerobic exercise (53, 128). However, in individuals with T2DM and cardiovascular complications, the feasibility and safety of HIIT may be limited by lower baseline fitness, reduced exercise tolerance, symptom burden, and the need for clinical supervision.

Beyond its prognostic value as a marker of cardiovascular and all-cause mortality risk, CRF also provides physiological information that is relevant to exercise prescription in T2DM. This is particularly important because adults with T2DM generally show lower absolute and relative VO_2_peak than non-diabetic controls (129). VO_2_peak reflects the integrated capacity for central oxygen delivery and peripheral oxygen extraction (129, 130). Therefore, the lower exercise capacity and exercise intolerance observed in T2DM may arise from different combinations of impaired cardiac output reserve, diastolic dysfunction, reduced skeletal-muscle perfusion or microvascular O_2_ delivery, and impaired peripheral O_2_ extraction or mitochondrial oxidative capacity (129–131). These central and peripheral abnormalities may also impair the dynamic VO_2_ response during transitions to moderate- or high-intensity exercise (131, 132). Such slowed VO_2_ kinetics reflects a delayed rise in oxidative metabolism relative to the energetic demands of contracting muscle and suggests impaired matching between O_2_ delivery and O_2_ utilization in T2DM (131, 132). Accordingly, these physiological constraints may amplify interindividual variability in exercise tolerance in T2DM, supporting the use of measured physiological and perceptual responses, rather than predicted values alone, to individualize exercise intensity.

BP control is another key therapeutic target. A recent network meta-analysis in individuals with T2DM suggested that exercise prescriptions for BP control may need to be tailored according to baseline BP stage. Resistance training appeared most suitable for elevated systolic BP, HIIT or aerobic exercise for early-stage hypertension, and mind-body exercise, such as yoga, tai chi, or qigong, for more advanced hypertension, with estimated optimal weekly doses of approximately 826 MET-min/week for systolic BP reduction and 994 MET-min/week for diastolic BP reduction (133). Dyslipidemia is also highly relevant in T2DM with cardiovascular complications. However, exercise training should be viewed as an adjunct to, rather than a substitute for lipid-lowering medication to achieve target values indicated by current guidelines (123). In addition to structured exercise, reducing and breaking up prolonged sedentary time may provide a practical complementary strategy for improving cardiometabolic health, because sedentary time is independently associated with higher CVD risk even among individuals meeting recommended physical activity targets (134, 135).

Safety and feasibility are particularly important when prescribing exercise to individuals with T2DM and cardiovascular complications. The ADA consensus report states that pre-exercise medical evaluation is not required for individuals without symptoms of CVD or microvascular complications (4). In contrast, the Chinese Society of Endocrinology recommends cardiovascular risk assessment for all patients with T2DM before an exercise prescription is initiated (136). Although the need for pre-exercise stress testing in asymptomatic adults with T2DM remains controversial, individuals with CVD symptoms or microvascular diseases should receive pre-exercise medical evaluation. Moreover, because resistance exercise could induce an inappropriate increase in BP, patients with hypertension should avoid static/isometric loading, forceful sustained gripping, and the Valsalva manoeuvre (137).

### 7.2 Cardiovascular autonomic neuropathy

Cardiovascular autonomic neuropathy (CAN) is an under-recognized but clinically important complication of T2DM, characterized by impaired autonomic regulation of heart rate (HR), vascular tone, and BP after other causes have been excluded (138, 139). Early CAN may be asymptomatic and detectable only through reduced heart rate variability (HRV), whereas progressive disease may manifest as resting tachycardia, a fixed HR, chronotropic incompetence, orthostatic hypotension, exercise intolerance, and silent myocardial ischemia (139, 140). In a contemporary community-based cohort of adults with T2DM, possible and definite CAN were identified in 33.7% and 15.3% of participants, respectively, and both categories were associated with a graded increase in all-cause mortality (141).

CAN may blunt the heart-rate, blood-pressure, and cardiac-output responses to exercise, so a low exercise HR does not necessarily indicate a low metabolic load (142, 143). Accordingly, age-predicted maximal heart rate should not be used as the sole basis for exercise prescription in patients with CAN or in those receiving HR-limiting medications (144–146). HR-based targets should be derived from an exercise test performed under the patient’s usual medication regimen and interpreted together with workload, perceived exertion, BP, and symptoms. In clinical practice, when feasible, exercise intensity should be anchored to symptom-limited exercise testing, preferably cardiopulmonary exercise testing, using the first and second ventilatory thresholds and their corresponding external workloads. These test-derived thresholds can then guide training prescription, with continuous aerobic exercise initiated near the first ventilatory threshold and progressively advanced toward the second ventilatory threshold according to exercise tolerance and clinical status (147).

Available evidence suggests that structured exercise may improve cardiac autonomic function in patients with T2DM and CAN, although current evidence is based mainly on surrogate autonomic outcomes rather than hard cardiovascular endpoints. A meta-analysis of 21 studies suggested that exercise training may improve HRV-based measures of cardiac autonomic function in patients with T2DM, with the most consistent evidence observed for supervised endurance training (148). A systematic review of randomized trials further indicated that combined training, HIIT, and progressive resistance training may improve cardiac autonomic function assessed by HRV, heart rate recovery, and baroreflex sensitivity, but emphasized that many studies were small and of limited methodological quality (149). Consistent with these reviews, randomized trials in patients with T2DM and CAN or abnormal HRR showed that combined training improved autonomic outcomes, including resting HR, HRR, and HRV, and was also associated with better glycemic control and exercise capacity (150, 151). Overall, exercise may be a useful adjunct for improving autonomic regulation and cardiometabolic health in clinically stable patients with CAN, but current evidence does not show that exercise reverses CAN or reduces CAN-related cardiovascular events or mortality.

### 7.3 Diabetic kidney disease

Chronic kidney disease (CKD) is defined as glomerular filtration rate (GFR) <60 mL/min/1.73 m² and/or albumin-to-creatinine ratio (ACR) ≥30 mg/g and/or other markers of kidney damage present (152). It has been estimated that 40% of people with diabetes will develop diabetic kidney disease (DKD) (153–156). The classic progression of DKD begins with glomerular hyperfiltration, followed by increased urinary albumin excretion and progressive decline in GFR, eventually leading to CKD (157). The prevalence of hyperfiltration in patients with T2DM is 6-73%, and hyperfiltration is associated with the development of more severe kidney damage in the later stages (154, 158).

Given the high burden of DKD in people with T2DM, regular physical activity is generally recommended as part of risk-factor management. Both the ADA Standards of Care and Kidney Disease Improving Global Outcomes guidelines recommend patients with diabetes and/or CKD undertake moderate-intensity physical activity for a cumulative duration of at least 150 min/week (5, 152). Although physical activity can acutely increase urinary albumin excretion, current evidence does not indicate that vigorous-intensity exercise accelerates CKD progression and specific exercise restrictions are generally unnecessary for most people with CKD (3, 5). Notably, patients with DKD may have greater muscle fatigability than those with T2DM alone, which may limit exercise adherence and necessitate individualized exercise prescription (159). However, current studies regarding the renal benefits of exercise in T2DM or DKD remain inconsistent.

Observational studies did not find a significant association between physical activity and lower DKD risk, although one reported correlations with intermediate kidney markers, including estimated GFR and ACR (160, 161). Recent randomized controlled trials provide some support for a potential renal benefit of exercise. In adults with T2DM without pre-existing kidney disease, both HIIT and combined training improved estimated GFR, but the latter did not significantly reduce urinary albumin excretion (162, 163). In patients with established DKD, exercise-based rehabilitation improved 6-min walk distance and several renal function markers (164). In another study, periodic resistance training appeared more effective than aerobic exercise in reducing ACR and improving GFR-related measures (165). In the Look AHEAD trial, an intensive lifestyle intervention combining caloric restriction with increased physical activity, targeting at least 175 min/week of moderate-intensity exercise, showed beneficial effects on kidney outcomes in participants aged ≥60 years, whereas similar benefits were not observed in younger participants (166, 167). Meta-analytic evidence remains uncertain. A meta-analysis in patients with diabetes suggested that physical activity was associated with improved renal function, reflected by increased GFR and reduced ACR, but was not significantly associated with a lower incidence of DKD (168). In contrast, a meta-analysis focusing on patients with established DKD found no significant association between exercise and renal function outcomes (169). Overall, these findings suggest physical activity may have beneficial effects on intermediate surrogate renal outcomes. However, the evidence remains constrained by small samples, short follow-up, heterogeneous exercise protocols, and surrogate endpoints. Whether exercise can independently prevent DKD onset or slow its long-term progression therefore remains unclear.

### 7.4 Diabetic retinopathy

Diabetic retinopathy (DR) is a major microvascular complication of T2DM and is a leading cause of blindness, affecting 34.6% of individuals with diabetes (170). Because DR is driven by chronic hyperglycemia, hypertension, dyslipidemia, inflammation, oxidative stress, endothelial dysfunction, and retinal neurovascular injury, regular exercise and physical activity should be viewed as part of DR risk management (170–172). Current evidence, derived mainly from observational physical activity studies, suggests that higher physical activity is associated with a lower risk of DR onset or progression. However, the strength of evidence remains limited, and causality has not been established.

A meta-analysis of 22 studies found that physical activity was associated with a lower risk of DR, with moderate-intensity activity showing the most favorable association, whereas comparable protective effects were not observed for low- or high-intensity activity (173). Prospective studies in adults with T2DM suggest that higher physical activity is independently associated with lower risks of incident DR and DR progression (174–176). Dose-response analyses further showed that the greatest benefit was observed with exercise performed at approximately five times per week and 180 min/week (176). Device-measured evidence also suggests that moderate or moderate-to-vigorous physical activity may prevent or delay the progression of DR, which is partly mediated by lower BMI. Notably, exercise between 6:00 a.m. and 12:00 p.m. was independently associated with lower DR risk, whereas late-night physical activity between 0:00 and 5:59 a.m. was identified as an independent risk factor (177). This observation is supported by prospective evidence showing that an evening chronotype is associated with a higher risk of incident or progressive DR in adults with T2DM (178). Direct exercise-intervention trials are relatively scarce. In a before-after clinical trial of 40 patients with non-proliferative DR, 12 weeks of supervised moderate-intensity aerobic exercise significantly reduced fasting blood glucose and central macular thickness (179). However, the before-after design and small sample size limit causal interpretation.

Overall, current evidence supports physical activity as a potentially protective adjunct in the management of DR, but it should not be interpreted as a disease-specific treatment. Importantly, exercise prescription should be individualized according to DR severity. Patients with mild to moderate non-proliferative DR can generally be encouraged to increase physical activity, whereas those with severe non-proliferative or proliferative DR should avoid vigorous exercise, powerlifting, breath-holding, jumping, jarring or head-down activities which may increase the risk of vitreous hemorrhage or retinal detachment (3, 5).

### 7.5 Diabetic peripheral neuropathy and foot risk

Diabetic peripheral neuropathy (DPN) is one of the most common and disabling complications of T2DM. It may affect sensory, motor, and autonomic nerves, resulting in neuropathic pain, loss of protective sensation, gait instability, and increased risk of diabetic foot ulceration (172, 180, 181). The ADA emphasizes that up to 50% of DPN cases may be asymptomatic, and unrecognized DPN may expose patients to injuries, diabetic foot ulcers, and amputation (140). Although glycemic optimization is essential for overall diabetes care, intensive glycemic control has only a small and non-significant effect on preventing clinical neuropathy in T2DM (182). Moreover, specific treatments that reverse the underlying nerve damage in diabetes are currently lacking (172). Therefore, in individuals with DPN or elevated foot risk, exercise should be viewed primarily as a strategy to improve modifiable downstream impairments, including muscle weakness, impaired balance, limited ankle mobility, and abnormal plantar loading.

A recent meta-analysis of randomized controlled trials reported that exercise improved neurological dysfunction in patients with diabetes, with larger effects observed after moderate-intensity and 8-week interventions (183). Other meta-analyses show that exercise, especially balance and foot-ankle training, can improve selected musculoskeletal and balance-related outcomes in DPN, including ankle range of motion, hallux and toe strength, lower-limb functional strength, gait speed, and clinical balance tests (184–186). Consistently, a recent umbrella review of 14 systematic reviews reported potential benefits of exercise for neuropathic symptoms and functional outcomes in DPN, but evidence remains heterogeneous and insufficient to confirm benefits for fall-risk reduction or diabetic foot ulcer prevention (180). Furthermore, a randomized controlled trial reported that foot-ankle exercise did not improve DPN symptoms or severity, but improved selected functional outcomes (187). Overall, exercise may improve selected functional and neuropathic outcomes in DPN, but the evidence remains insufficient to establish whether these improvements translate into long-term clinical protection.

In DPN, a central safety concern is whether weight-bearing activity increases the risk of diabetic foot ulceration. This concern arises because repetitive vertical and shear stress on an insensate neuropathic foot can promote callus formation, subcutaneous hemorrhage, and ultimately ulceration (188). A recent systematic review found that 8-12-week foot-ankle exercise programs did not increase or decrease foot ulceration, pre-ulcerative lesions, adverse events, or barefoot peak plantar pressure during walking, although the certainty of evidence is low (189). Consistently, current International Working Group on the Diabetic Foot guidance recommends a risk-stratified approach: people at low or moderate ulcer risk may undertake foot-ankle exercise and gradually increase walking-related weight-bearing activity, whereas mechanically loaded foot-related exercises should be avoided in those with pre-ulcerative lesions or active ulcers because safety evidence is insufficient (190).

## 8 Age-specific considerations for exercise in T2DM

### 8.1 Older adults with T2DM

Diabetes represents a major and growing health burden in ageing populations. In 2024, the estimated global prevalence of diabetes among adults aged 65–99 years was 23.7% (1). Older adults with diabetes are more likely to experience functional disability, accelerated skeletal muscle loss, mobility impairment, frailty, and multimorbidity than those without diabetes (31). Joint mobility is also restricted in this population, as age-related collagen glycation and connective-tissue stiffening are amplified by chronic hyperglycemia, thereby promoting advanced glycation end product-mediated collagen cross-linking in joint capsules, ligaments, and muscle-tendon units (191, 192).

Current guidelines and consensus statements recommend flexibility and balance training 2–3 times/week for older adults with diabetes to improve the range of motion around joints and reduce the risk of falls, respectively. Evidence regarding stretching as a stand-alone intervention remains limited. A meta-analysis suggested that stretching may improve blood glucose and HbA1c in individuals with T2DM, but the findings were constrained by substantial heterogeneity (I² = 96.56%) and variable study quality (193). Increasing evidence suggests that structured exercise programs incorporating flexibility and balance as major components may offer broader metabolic and functional benefits in this population. A meta-analysis of 11 randomized controlled trials involving 944 older adults with diabetes showed that traditional Chinese exercise reduced fasting blood glucose, HbA1c, and BMI, while a meta-analysis of randomized trials in adults with T2DM found that Tai Chi significantly reduced fasting blood glucose and HbA1c (194, 195). Pilates has emerged as a potentially feasible adjunct exercise modality for older adults with T2DM. In a systematic review of 11 randomized trials, HbA1c reductions were frequently reported within Pilates groups, whereas only one trial showed a greater HbA1c reduction compared with controls (196). More directly, a recent randomized trial in older adults with T2DM reported that a 12-week Pilates program significantly improved capillary blood glucose, handgrip strength, gait speed, balance, and flexibility compared with usual care (197). Yoga-based evidence is less specific to older adults, but a recent randomized trial in women with T2DM showed that 12 weeks of yoga improved fasting glucose and HbA1c (198). Overall, these findings support flexibility- and balance-oriented exercise as a useful adjunct in older adults with T2DM, particularly when therapeutic goals extend beyond glycemic control to mobility, balance, and functional independence.

Diabetes in older adults is associated with reduced muscle strength, impaired muscle quality, and accelerated loss of muscle mass, which may further increase the risk of sarcopenia, dynapenia, and osteopenia (31). Accordingly, in older adults with T2DM, the clinical relevance of resistance training should not be viewed solely as a metabolic intervention for improving glycemic control and insulin sensitivity, but also as a key therapeutic strategy for preserving muscle strength, muscle mass, and physical function. Current evidence generally supports the beneficial effects of resistance training on glycemic control in older adults with T2DM, with particularly consistent improvements observed in muscle strength and function-related outcomes (34, 35, 199, 200). Nevertheless, conventional resistance training may not be appropriate for all older adults with T2DM, particularly those with frailty, sarcopenia, joint pain, or reduced exercise tolerance. Therefore, low-load and functionally adapted resistance-training strategies have attracted increasing attention. Despite imposing substantially lower mechanical loads, low-load BFR-RT can provide a robust muscular stimulus and may induce improvements in muscle strength and hypertrophy comparable to conventional higher-load resistance training (45–48). Another feasible strategy of low-load resistance exercise is elastic-band resistance training. Recent studies suggest that elastic-band resistance training may improve glycemic control, muscle function, and frailty status in older adults with diabetes (201–204).

### 8.2 Youth-onset T2DM

Traditionally, T2DM has been regarded primarily as a disease of middle-aged and older adults and was considered relatively uncommon in children and adolescents. However, over the past two decades, the incidence and prevalence of youth-onset T2DM have increased substantially across multiple regions, making it an increasingly important public health concern (205–208). Importantly, youth-onset T2DM should not be viewed simply as adult T2DM occurring earlier in life. Compared with adult-onset disease, it is characterized by a more aggressive clinical trajectory, including accelerated β-cell dysfunction, greater insulin resistance, poorer therapeutic responsiveness, and earlier development of diabetes-related complications (209, 210). These features prolong lifetime exposure to hyperglycemia and cardiometabolic risk, underscoring the need for early, developmentally tailored intervention strategies. Current guidelines recommend that children and adolescents with T2DM engage in 60 min/day of moderate-to-vigorous physical activity, primarily aerobic, with vigorous-intensity aerobic activities and muscle-strengthening and bone-strengthening activities incorporated at least 3 days per week (211, 212).

However, direct intervention evidence supporting specific exercise prescriptions for youth-onset T2DM remains scarce. Observational evidence suggests that higher physical activity frequency is associated with lower HbA1c in adolescents with T2DM, with no significant differences in pharmacological treatment regimens across activity groups (213). A recent systematic review identified only three intervention studies targeting physical activity alone in children and adolescents with T2DM, and these studies did not provide convincing evidence for an HbA1c-lowering effect (214). In a small supervised exercise trial, 12 weeks of combined training improved vascular function and muscular strength in adolescents with T2DM, but did not significantly improve insulin sensitivity, BMI, or CRF, with vascular benefits not sustained after detraining (215). Low adherence represents an additional barrier in youth-onset T2DM. In a 16-week personalized exercise intervention, adolescents with T2DM showed lower adherence than adolescents with obesity alone and reported greater barriers to exercise participation (216).

## 9 Clinical implications and future directions

**Figure 2:**
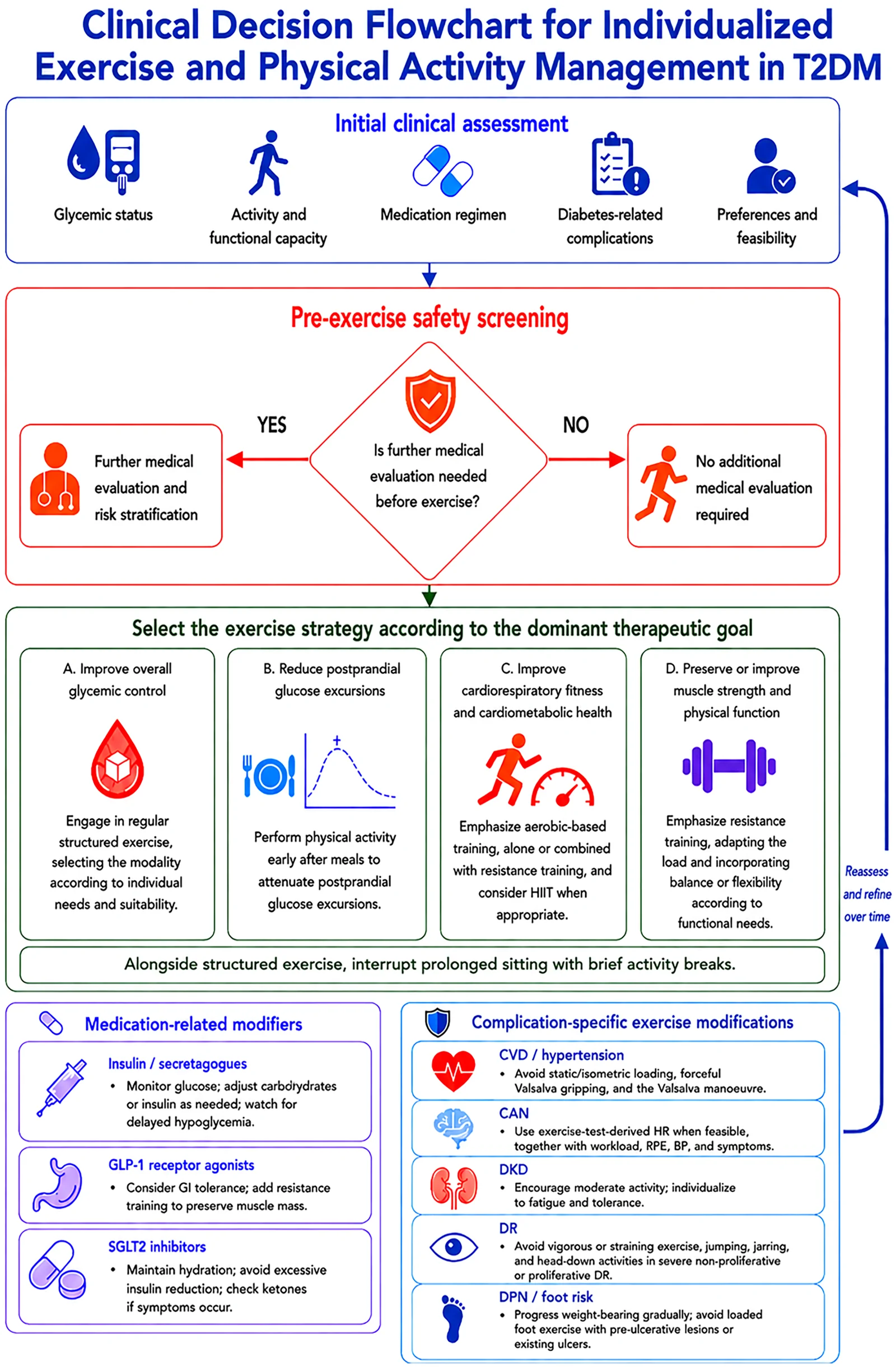
Clinical decision flowchart for individualized exercise and physical activity management in T2DM. The flowchart outlines a practical approach to individualized exercise management in T2DM. It begins with assessment of glycemic status, activity and functional capacity, medication regimen, diabetes-related complications, and patient preferences and feasibility, followed by pre-exercise safety screening and further medical evaluation when indicated. Exercise strategies are then selected according to the dominant therapeutic goal. Breaking up prolonged sitting with brief activity breaks is incorporated as a complementary strategy. Exercise prescription is further adapted according to medication-related considerations and diabetes-related complications, with periodic reassessment and refinement based on clinical response, safety, functional capacity, and feasibility.

Taken together, current evidence supports a more individualized and clinically oriented approach to exercise and physical activity management in T2DM (**Figure 2**). Building on existing guideline recommendations, this framework integrates clinically relevant factors to guide the context-specific adaptation of exercise prescriptions in T2DM care. First, exercise prescription should build on guideline-recommended exercise targets while allowing adaptation to therapeutic goals and patient characteristics (**Table 1**). Although at least 150 minutes/week of moderate-intensity aerobic exercise is a practical foundational recommendation for most patients, the exercise regimen should be tailored according to the patient’s primary therapeutic goals, baseline fitness, comorbidities, functional status, and personal preferences. Second, exercise prescription for patients with T2DM should not be limited to exercise modality, intensity, and weekly volume, but should also incorporate exercise timing and sedentary behavior into the overall management framework. Postprandial activity, particularly activity performed early after meals, may represent a clinically practical strategy for attenuating postprandial glucose excursions. Meanwhile, interrupting prolonged sitting with brief activity breaks should be incorporated as a complementary sedentary-behavior strategy alongside structured exercise and habitual physical activity management in T2DM. In selected adults with T2DM, particularly those with marked glycemic variability or a high risk of hypoglycemia, CGM may offer a practical approach to individualizing exercise prescriptions by identifying short-term glucose excursions and guiding adjustments in exercise timing, intensity, and safety monitoring. Third, diabetes-related complications should not be simply viewed as contraindications to exercise. Instead, they should be understood as clinical factors that require risk stratification and prescription modification. For patients with diabetes-related complications, exercise prescription should be guided by complication severity, clinical status, and exercise-related risk, with medical evaluation considered for those with advanced complications. Fourth, age-specific factors should also be incorporated into clinical decision-making for exercise management in T2DM. For older adults with T2DM, the goals of exercise intervention should not be limited to glycemic control, but should also include preservation of muscle strength, balance, joint mobility, and functional independence. In contrast, youth-onset T2DM is characterized by more rapid disease progression and longer lifetime exposure to hyperglycemia and cardiometabolic risk. Exercise management in this population should therefore emphasize early intervention and the establishment of long-term healthy behaviors.

**Table 1:** Summary of recommended and estimated exercise doses in T2DM.

Exercise modality/context	Dose category	Exercise dose	Therapeutic target	Interpretation
Aerobic training	Guideline recommendation	≥150 min/week of moderate- to vigorous-intensity aerobic activity	Glycemic control and cardiometabolic health	Standard weekly aerobic training target recommended by guidelines.
Resistance training	Guideline recommendation	2–3 sessions/week on nonconsecutive days at moderate- or vigorous-intensity	Glycemic control, muscular strength, and physical function	Standard weekly resistance training target recommended by guidelines.
Combined training	Estimated minimum effective dose	~370 MET-min/week	HbA1c	Estimated minimum weekly dose associated with significant HbA1c reduction.
Combined training	Estimated optimal dose	~1030 MET-min/week	HbA1c	Estimated weekly dose associated with the greatest HbA1c reduction.
HIIT	Guideline recommendation	75 min/week	Glycemic control and cardiometabolic health	Shorter-duration option for more physically fit adults.
HIIT	Estimated minimum effective dose	~330 MET-min/week	HbA1c	Estimated minimum weekly dose associated with significant HbA1c reduction.
Overall exercise	Estimated minimum effective dose	~840 MET-min/week	HbA1c	Estimated minimum weekly dose associated with significant HbA1c reduction.
Overall exercise	Dose-response attenuation point	~1300 MET-min/week	HbA1c	Approximate dose beyond which additional HbA1c benefit is attenuated.
Overall exercise	Estimated optimal dose	~1100 MET-min/week	HbA1c	Estimated optimal weekly dose for HbA1c reduction across baseline HbA1c categories.
Blood pressure control	Estimated optimal dose	~826 MET-min/week	Systolic blood pressure	Estimated optimal weekly dose for maximum systolic blood pressure reduction.
Blood pressure control	Estimated optimal dose	~994 MET-min/week	Diastolic blood pressure	Estimated optimal weekly dose for maximum diastolic blood pressure reduction.
DKD	Guideline recommendation	≥150 min/week of moderate-intensity exercise	General DKD risk management	Guideline-recommended weekly physical activity target, adjusted to cardiovascular and physical tolerance.
DR	Observational optimal-dose estimate	~5 sessions/week; ~180 min/week	DR incidence	Approximate frequency and duration associated with the lowest observed DR incidence.
Youth-onset T2DM	Guideline recommendation	60 min/day of moderate-to-vigorous, primarily aerobic activity; vigorous aerobic and muscle- and bone-strengthening activities ≥3 days/week.	Cardiometabolic health and physical fitness	Standard daily physical activity target recommended by pediatric diabetes guidelines.

Future research should focus on developing clinically applicable approaches to individualized exercise prescription and determining how prescriptions can be adjusted over time according to treatment response, tolerance, and safety. Particular attention should be given to older adults, individuals with youth-onset T2DM, and patients with diabetes-related complications, whose therapeutic goals, clinical status, and exercise capacity may differ substantially. Medication–exercise interactions also require further clarification because they may alter both treatment response and exercise-related risk. In addition, future studies should clarify the biological basis of heterogeneity in exercise response. Genetic variation, gut microbiome composition, and baseline metabolic profiles may influence individual responses to exercise (217). Candidate biomarkers, including circulating microRNAs, metabolic signatures, inflammatory markers, and microbiome-derived metabolites, should be evaluated as potential predictors of individual benefit from exercise interventions (218, 219). Integrating multi-omics profiling with standardized exercise trials may provide a biological basis for future precision exercise prescription in T2DM.

## 10 Conclusion

Exercise is a core component of T2DM management, with benefits for glycemic regulation, physical function, and cardiometabolic health. Exercise prescription in T2DM should build on established guideline recommendations while incorporating individualized and risk-stratified adaptations. Exercise modality and dose should be selected according to therapeutic goals, baseline fitness, comorbidities, safety, and feasibility. Exercise timing should be considered when designing structured exercise programs, as it may influence metabolic responses. Beyond planned exercise sessions, interrupting prolonged sitting should be viewed as a complementary behavioral target for improving glycemic control, insulin sensitivity, and cardiometabolic health. For individuals with diabetes-related comorbidities, older adults with T2DM, and those with youth-onset T2DM, prescriptions should be individualized to optimize metabolic and functional benefits while ensuring safety, matching functional capacity, and supporting long-term adherence. Whereas current ADA/ACSM recommendations provide foundational exercise targets and principles for individualized implementation, and existing precision-exercise frameworks primarily emphasize interindividual variability in exercise responses and its potential determinants, the present review integrates therapeutic goals, exercise timing, sedentary behavior, medication–exercise interactions, complication-specific considerations, functional capacity, and age-related factors to refine context-specific exercise prescription in T2DM.
