# Supplementary material for: Exercise and physical activity interventions in type 2 diabetes mellitus
Source: Front Endocrinol (Lausanne). 2026 Aug 20;17:1887543. doi: 10.3389/fendo.2026.1887543 (PMC13539497; doi:10.3389/fendo.2026.1887543)
Supplement: Supplementary file 1 [file DataSheet1.pdf]

Supplementary Table 1. Search strategy

Database	PubMed
Date searched	2 April 2026
Date limits	No publication-date restrictions were applied.
Restrictions	No language restrictions were applied
Search for type 2 diabetes and exercise	(("Diabetes Mellitus, Type 2"[Mesh]) OR (Diabetes Mellitus, Type II[Title/Abstract]) OR (Type 2 Diabetes[Title/Abstract]) OR (Diabetes, Type 2[Title/Abstract]) OR (Type 2 Diabetes Mellitus[Title/Abstract])) AND (("Exercise"[Mesh]) OR (Exercises[Title/Abstract]) OR (Exercise Training[Title/Abstract]) OR (Exercise Therapy[Title/Abstract]) OR (Acute Exercise[Title/Abstract]) OR (Physical Activity[Title/Abstract]) OR (Exercise modality[Title/Abstract]) OR (Aerobic training[Title/Abstract]) OR (Resistance training[Title/Abstract]) OR (Combined training[Title/Abstract]) OR (High-intensity interval training[Title/Abstract]) OR (HIIT[Title/Abstract]) OR (Exercise dose[Title/Abstract]) OR (Exercise intensity[Title/Abstract]) OR (Exercise timing[Title/Abstract]) OR (Postprandial exercise[Title/Abstract]) OR (Activity breaks[Title/Abstract]) OR (Breaking up sitting[Title/Abstract]) OR (Sedentary Behavior[Title/Abstract]))
Supplementary search for type 2 diabetes, exercise, and diabetes-related complications	(("Diabetes Mellitus, Type 2"[Mesh]) OR (Diabetes Mellitus, Type II[Title/Abstract]) OR (Type 2 Diabetes[Title/Abstract]) OR (Diabetes, Type 2[Title/Abstract]) OR (Type 2 Diabetes Mellitus[Title/Abstract])) AND (("Exercise"[Mesh]) OR (Exercises[Title/Abstract]) OR (Exercise Training[Title/Abstract]) OR (Exercise Therapy[Title/Abstract]) OR (Acute Exercise[Title/Abstract]) OR (Physical Activity[Title/Abstract]) OR (Exercise modality[Title/Abstract]) OR (Aerobic training[Title/Abstract]) OR (Resistance training[Title/Abstract]) OR (Combined training[Title/Abstract]) OR (High-intensity interval training[Title/Abstract]) OR (HIIT[Title/Abstract]) OR (Exercise dose[Title/Abstract]) OR (Exercise intensity[Title/Abstract])) AND (("Diabetes Complications"[Mesh]) OR (Diabetes complications[Title/Abstract]) OR (Cardiovascular disease[Title/Abstract]) OR (Cardiometabolic risk[Title/Abstract]) OR (Diabetic kidney disease[Title/Abstract]) OR (Chronic kidney disease[Title/Abstract]) OR (Diabetic nephropathy[Title/Abstract]) OR (Diabetic retinopathy[Title/Abstract]) OR (Diabetic peripheral neuropathy[Title/Abstract]) OR (Diabetic neuropathy[Title/Abstract]) OR (Diabetic foot[Title/Abstract]) OR (Foot ulcer[Title/Abstract]))

Database	Web of Science
Date searched	2 April 2026
Date limits	No publication-date restrictions were applied.
Restrictions	No language restrictions were applied
Search for type 2 diabetes and exercise	(TS=(Diabetes Mellitus, Type 2) OR AB=(Diabetes Mellitus, Type II OR Type 2 Diabetes OR Diabetes, Type 2 OR Type 2 Diabetes Mellitus)) AND (TS=(Exercise) OR AB=(Exercises OR Exercise Training OR Exercise Therapy OR Acute Exercise OR Physical Activity OR Exercise modality OR Aerobic training OR Resistance training OR Combined training OR High-intensity interval training OR HIIT OR Exercise dose OR Exercise intensity OR Exercise timing OR Postprandial exercise OR Activity breaks OR Breaking up sitting OR Sedentary Behavior))
Supplementary search for type 2 diabetes, exercise, and diabetes-related complications	(TS=(Diabetes Mellitus, Type 2) OR AB=(Diabetes Mellitus, Type II OR Type 2 Diabetes OR Diabetes, Type 2 OR Type 2 Diabetes Mellitus)) AND (TS=(Exercise) OR AB=(Exercises OR Exercise Training OR Exercise Therapy OR Acute Exercise OR Physical Activity OR Exercise modality OR Aerobic training OR Resistance training OR Combined training OR High-intensity interval training OR HIIT OR Exercise dose OR Exercise intensity)) AND (TS=(Diabetes Complications) OR AB=(Diabetes complications OR Cardiovascular disease OR Cardiometabolic risk OR Diabetic kidney disease OR Chronic kidney disease OR Diabetic nephropathy OR Diabetic retinopathy OR Diabetic peripheral neuropathy OR Diabetic neuropathy OR Diabetic foot OR Foot ulcer))
